# Outcomes and Toxicity of Stereotactic Radiotherapy in Non-small Cell Lung Cancer Patients With Interstitial Lung Disease: A Multicentre Cohort Study

**DOI:** 10.7759/cureus.100124

**Published:** 2025-12-26

**Authors:** Alexander J Sherlock, Annalise M Tanaka, Harry E Daniels, Antony Carver, Steven Watkins, Sundus Yahya

**Affiliations:** 1 Hospital Medicine, Queen Elizabeth Hospital Birmingham, Birmingham, GBR; 2 Immunology, Queen Elizabeth Hospital Birmingham, Birmingham, GBR; 3 Cancer Center, Queen Elizabeth Hospital Birmingham, Birmingham, GBR

**Keywords:** ild interstitial lung disease, lung sabr, non-small cell lung carcinoma (nsclc), radiation-induced toxicity, radio oncology

## Abstract

Background

Lung cancer remains a major cause of cancer-related mortality, with non-small cell lung cancer (NSCLC) comprising most cases. For early-stage NSCLC (ES-NSCLC), surgery is curative but often unsuitable. Stereotactic ablative radiotherapy (SABR) offers curative intent, achieving high local control with generally acceptable toxicity. Yet, patients with interstitial lung disease (ILD) face increased risks, including high-grade and fatal toxicity. ILD-adapted dose and fractionation have been introduced, as evaluated in studies such as ASPIRE-ILD. We evaluated real-world outcomes of SABR using modified fractionation in ES-NSCLC patients with ILD.

Methods

A multicentre retrospective cohort study was conducted across four UK hospitals (February 2020-May 2025). Consecutive patients with stages I-II NSCLC, confirmed ILD on high-resolution computed tomography (HRCT) or pathology, and treated with ILD-adapted SABR were included. Clinical, radiologic, pulmonary function, and dosimetric data were extracted. The primary outcome was treatment-related toxicity; secondary outcomes included overall survival (OS), progression-free survival (PFS), time to progression, and local control. Kaplan-Meier analysis estimated survival. Subgroup analyses explored associations with pulmonary function, ILD subtype, Eastern Cooperative Oncology Group (ECOG) performance status, biologically effective dose (BED), and fractionation schedule.

Results

Eleven patients (median age 79.9, 54.5% male) were included. ILD subtypes were idiopathic interstitial pneumonia (45.5%), ILD with known cause (36.4%), and unclassifiable ILD (18.2%). ECOG was 0-1 in 27.3%, 2 in 45.5%, and 3 in 27.3%. Median diffusing capacity for carbon monoxide (DLCO) was 70% predicted; one patient had DLCO <40%. Most received five-fraction SABR (72.7%). All patients experienced toxicity (median onset three months); grades 1-2 affected 81.8% of patients, and grade ≥3 events occurred in 36.4%. This included three treatment-related deaths (27.3%): pneumonitis, pneumonia, or progressive fibrosis. Median follow-up was 21.9 months. Median PFS was 8.6 months, and median OS was 16.3 months, with one-year OS of 60.6%. Local control was achieved in 90% of first planned imaging. Progression included regional nodal relapse (n = 3) and distant metastasis (n = 1). Subgroup analyses showed no significant associations between DLCO, ECOG, ILD subtype, BED, or fractionation and grade ≥3 toxicity or survival, though toxicity rates varied: 40% in idiopathic interstitial pneumonia, 25% in ILD with known cause, and 50% in unclassifiable ILD.

Discussion and conclusion

In this real-world cohort of patients with ES-NSCLC and ILD treated with modified SABR, treatment was feasible and achieved encouraging local control and median OS. However, toxicity was substantial, with grade ≥3 events in over one-third and three treatment-related deaths. No significant associations were identified between severe toxicity and DLCO, ILD subtype, BED, or performance status, though the small sample limited statistical power. This study provides real-world evidence supporting SABR as a potential option in selected ILD patients but emphasises the need for careful risk stratification and early monitoring. Outcomes highlight the challenges of applying trial protocols to physiologically impaired, heterogeneous ILD populations. Larger prospective studies are required to validate these findings and develop refined, ILD-specific SABR frameworks.

## Introduction

Lung cancer is the leading cause of cancer-related mortality globally, with non-small cell lung cancer (NSCLC) accounting for approximately 85% of all lung cancer cases [1,2]. The burden of lung cancer remains substantial, with an estimated 2,480,700 new cases and over 1,817,500 deaths annually worldwide, with a 5-year survival rate of just 10-33% across all stages [3,4]. Early-stage NSCLC (ES-NSCLC) typically refers to stages I and II disease, in which the tumour is confined to the lung and, in some cases, involves limited regional lymph nodes, but without distant metastasis [5]. A curative approach may be surgical resection, though many patients are too comorbid.

Stereotactic ablative body radiotherapy (SABR) has emerged as the preferred alternative in patients deemed inoperable [6]. SABR delivers high-dose, targeted radiation in a limited number of fractions, with minimal exposure to surrounding tissue. Guidelines from the National Comprehensive Cancer Network (NCCN) recommend SABR not only for inoperable patients, but also increasingly support its use in operable patients who refuse surgery, or for whom surgery poses a significant risk [7,8]. SABR provides acceptable overall survival (OS) and progression-free survival (PFS) with multiple prospective and retrospective studies reporting local control rates exceeding 68-95% at three years and OS of 37% at three years. Timmerman et al. reported a three-year local control rate of 98% in a phase II study involving inoperable ES-NSCLC patients, along with an OS rate of 55.8% at 3 years [6,9,10]. These outcomes are particularly significant given the generally poor prognosis of untreated ES-NSCLC in inoperable patients. Importantly, SABR achieves these outcomes with minimal invasiveness and a relatively low toxicity profile.

However, risks, such as radiation pneumonitis, rib fractures, and chest wall pain, must be acknowledged and mitigated through careful planning and adherence to dose constraints [11-13]. Additional non-pulmonary toxicities also need to be considered, such as fatigue and dermal reactions. Studies have reported rates of severe toxicities as high as 16-27%, with pneumonitis being the most frequent grade 3 or greater toxicity (Common Terminology Criteria for Adverse Events (CTCAE) v5.0) [7,14,15]. Some have continued further, describing central tumour location, large planning target volume, and large gross target volume as prognostic factors for these severe toxicities [7]. Despite these potential adverse effects, the high rates of tumour control, favourable survival outcomes, and improved quality of life have positioned SABR as a frontline treatment for ES-NSCLC.

Interstitial lung disease (ILD) refers to a heterogeneous group of pulmonary disorders characterised by inflammation and fibrosis of the lung interstitium, leading to impaired gas exchange and progressive respiratory dysfunction. Common ILD subtypes include usual interstitial pneumonia (UIP), exposure-related ILD, and unclassifiable ILD [16]. NSCLC patients with pre-existing ILD are at elevated risk for SABR-related toxicity, particularly radiation pneumonitis(RP), which may manifest more severely and with higher mortality than in patients without ILD [17,18]. This increased susceptibility is likely due to the underlying fibrotic and inflammatory milieu present in ILD, which shares pathogenic features with RP, as well as their shared trigger of epithelial injury [19]. SABR in those with ILD presents a heightened risk of radiation-induced lung injury, including grade 5 (CTCAE) toxicities [17]. To reduce this risk, several measures have been developed and increasingly implemented in clinical practice. Key strategies include modification of the radiation dose and fractionation, careful patient selection, and the use of advanced treatment planning techniques. Reduced total doses and more protracted fractionation schedules (e.g., five or more fractions rather than three) have been employed to lower the biologically effective dose (BED) to surrounding lung tissue, thereby decreasing the risk of RP [20,21].

The phase II, non-randomised control trial ASPIRE-ILD demonstrated that SABR can provide meaningful survival benefit in those with ES-NSCLC and ILD with acceptable rates of serious toxicity, by applying ILD-adapted dose constraints and conservative dose delivery. The trial yielded results of 79% one-year OS, 74% one-year PFS, 92% two-year local control, and only 8% grade 5 toxicities, with the risk of grades 3-5 toxicities at half of those reported in a 2017 systematic review [18,20]. Palma et al. summarise that, “the toxic effect rates observed in [the] trial are within the range accepted in oncology in the delivery of curative-intent treatment” [20]. However, on balance, alternative therapies to SABR should also be considered after careful consideration of the individual patient. We have selected ASPIRE-ILD as an important comparator due to its providing novel, prospective data in a patient population historically cautiously approached with SABR. In light of the evidence published by ASPIRE-ILD, we have subsequently conducted what is, to our knowledge, the first real-world cohort analysis of patients with both ES-NSCLC and ILD treated with SABR using modified fractionation protocols.

## Materials and methods

Study design and setting

This investigation was conducted as a multicentre, retrospective cohort study. Hospitals included were the Queen Elizabeth Hospital Birmingham, a large tertiary referral centre, Solihull, Worcester, and Good Hope Hospital, acute general hospitals. The investigation was designed to evaluate treatment-related toxicities and clinical outcomes among patients with stages I-II NSCLC and coexisting ILD, treated with SABR using modified lower dose regimens based on ASPIRE-ILD trial recommendations [20]. A retrospective design was chosen to enable real-world assessment of outcomes in a high-risk patient population, where prospective randomised trials may be impractical due to ethical or logistical barriers.

Patients were identified using institutional electronic medical records and radiotherapy planning databases. Diagnostic confirmation and treatment decisions were standardised through multidisciplinary tumour board review. Clinical, radiological, spirometric, and dosimetric data were retrospectively extracted and linked to clinical outcomes. The analysis included all patients who met eligibility criteria from February 2020 to May 2025.

Although retrospective in nature, the study adhered to established reporting standards for observational research, including the Strengthening the Reporting of Observational Studies in Epidemiology (STROBE) and Declaration of Helsinki guidelines [22,23]. 

Participants

Eligible patients met the following criteria: 1) radiologic or histologic confirmation of stage I-II NSCLC as per the 8th edition TNM classification [5], 2) coexisting ILD confirmed on high-resolution computed tomography (HRCT) or pathology where available, 3) treatment with SABR incorporating modified fractionation schemes based on ASPIRE-ILD trial dose constraints, 4) availability of pre-treatment pulmonary function test (PFT) data and post-treatment imaging [20].

Variables

The primary outcome was treatment-related toxicity, graded using CTCAE v5.0 [15]. Secondary outcomes included median OS, median PFS, estimated percentage of patients alive at one year, time to disease progression, and local tumour control. Local control was defined as the absence of radiographic progression within the treated volume at planned imaging six months post-SABR. Radiographic response criteria used was RECIST 1.1 [24].

Predictor variables included pre-treatment pulmonary function parameters, including forced expiratory volume in one second (FEV1), forced vital capacity (FVC), and diffusion capacity of carbon monoxide (DLCO). Dosimetric variables were expressed as BED using an α/β ratio of 3 Gy, which is appropriate for modelling normal lung tissue toxicity (e.g., pneumonitis). For tumour control modelling, using the linear quadratic model, BED was calculated using an α/β ratio of 10 Gy (BED 10). Clinical predictors included ILD subtype (e.g., UIP), Eastern Cooperative Oncology Group (ECOG) performance status, and SABR fractionation schedule [25].

Formal adjustment for confounders was not performed due to the small sample size, which limited the feasibility of multivariable modelling. However, subgroup analyses were performed for clinically relevant variables.

Data sources and measurement

Pulmonary function test data (FEV1, FVC, DLCO) were extracted from pre-treatment spirometry that was conducted in accordance with American Thoracic Society (ATS)/European Respiratory Society (ERS) standards [26]. ILD subtype classification was based on interpretation of HRCT scans by radiologists, with histopathologic confirmation incorporated when available, per international guidelines [27]. ILD severity was calculated by the ILD Gender-Age-Physiology (GAP) index [28].

Geometric and dosimetric parameters were obtained from the treatment planning system used for SABR. Organs-at-risk (OARs) were contoured according to the UK SABR Consortium Guidelines [29]. Full OAR constraints can be found in Appendix A and Appendix B. Normal tissue constraints were used from the UK 2022 Consensus Guidelines [30] and the ASPIRE-ILD trial [20]. The internal target volume (ITV) was derived from the union of gross tumour volume (GTV) outlines on individual phases of a 10-phase four-dimensional CT. As per UK SABR guidelines, a normal lung was defined as total lung minus GTV. The normal lung dose was summarised using the mean dose (Dmean) and V20.

SABR dose and fractionation were primarily guided by the UK SABR Consortium Guidelines [29], including considerations such as the proximity of the target to central structures. Clinical decision-making was further informed by ASPIRE-ILD trial [20] recommendations and individual consultant clinical oncologist judgment. ILD-specific adaptations were based on a comprehensive assessment incorporating tumour location, comorbidity burden, ILD severity as reflected by the GAP index [28], and the contemporary evidence base.

Toxicity grading was retrospectively determined from clinical notes and radiology reports, using CTCAE v5.0 criteria [15]. OS and PFS were calculated from the date of the final SABR fraction. Death was confirmed via hospital records or death certificates and discussed within a consultant-led multidisciplinary team to confirm attribution of death to treatment-related toxicity.

Local control was evaluated via serial computed tomography (CT) and/or positron emission tomography-computed tomography (PET-CT) interpreted through multidisciplinary radiologic review according to predefined radiographic criteria [5].

Bias

Selection bias was minimised by including all consecutive patients with stages I-II NSCLC and radiologic or histologic evidence of ILD who underwent SABR during the defined study period. All of whom were treated using modified dose constraints based on ASPIRE-ILD recommendations [20], without exclusions based on outcomes or comorbidity severity.

Information bias was reduced by adhering to standardised institutional protocols for pulmonary function testing, imaging interpretation, and toxicity grading. Data sources included structured electronic medical records, radiotherapy planning systems, and multidisciplinary team-reviewed imaging, which collectively increased internal validity.

While multivariable modelling was not feasible, confounding was partially addressed through stratified analyses based on DLCO thresholds, a known prognostic factor in both ILD and early-stage lung cancer populations [31,32].

Statistical methods

Baseline characteristics and treatment variables were summarised using descriptive statistics, including medians and interquartile ranges for continuous data, and frequencies for categorical variables. OS and PFS were estimated using the Kaplan-Meier method, with time-to-event calculated from the date of the final SABR fraction to the event of interest or last follow-up.

Subgroup analyses were conducted based on pre-specified clinical stratifiers, including DLCO (<40% vs. ≥40%), performance status (ECOG 0-2, ≥3), BED, and ILD subtype. The association between subgroup characteristics and the occurrence of toxicities was evaluated using Fisher’s exact test, appropriate for categorical data with small cell counts. Kaplan-Meier analysis was used to estimate survival.

No imputation was necessary, as there was no missing data for the primary variables of interest.

Loss to follow-up or matching adjustments were not applicable in this single-arm retrospective cohort study.

Sensitivity analyses were not conducted due to the small cohort size. 

Study size

All eligible patients with ES-NSCLC and ILD who received SABR between February 2020 and May 2025 were included, reflecting a real-world design suited to rare and high-risk patient subgroups. 

Quantitative variables

Key continuous variables included pulmonary function indices (DLCO, FEV1, FVC), geometric parameters (PTV), and dosimetric parameters (lung V20, Dmean). These were analysed as both continuous values and categorised when clinically relevant. For example, DLCO was dichotomised at 40% predicted, based on established toxicity risk thresholds [33].

Descriptive statistics summarised the quantitative data. Associations with clinical outcomes were evaluated using non-parametric tests, given the small sample size and non-normal distributions.

## Results

Participants

Between February 2020 and May 2025, a total of 13 patients with ES-NSCLC and ILD were identified from institutional radiotherapy databases. After applying the eligibility criteria, two patients were excluded due to the absence of radiographic evidence of ILD on HRCT. The final cohort comprised 11 patients who received SABR and were included in the analysis.

All patients were labelled “Patient 1-11” and, for continuity, were referred to solely by their assigned number.

All 11 patients had complete outcome data available. Ten patients (90.9%) completed the planned SABR course, while one patient (9.1%) discontinued treatment after two fractions due to acute toxicity. Survival status and toxicity outcomes were confirmed through hospital records and electronic documentation.

Descriptive data

The cohort included 11 patients with a median age of 79.9 years (73.3-90.0) at the time of initiation of radiotherapy. All patients had a confirmed diagnosis of stage I-II NSCLC and radiological evidence of ILD on HRCT. Most patients were male (n = 6, 54.5%). Median ILD GAP index was 3.0 (2.0-5.0).

The ILD subtype distribution was as follows:

By Major ATS/ERS Category

Idiopathic interstitial pneumonia (IIP) was observed in five patients (45.5%). ILD with a known cause or association was present in four patients (36.4%). Unclassifiable ILD was identified in two patients (18.2%).

By Detailed Subtype

Exposure-related ILD (asbestos-related) was observed in two patients (18.2%). Connective tissue disease-associated ILD related to rheumatoid arthritis (CTD-ILD (RA)) with a UIP pattern was present in two patients (18.2%). Idiopathic pulmonary fibrosis (IPF) with a UIP pattern, including the combined pulmonary fibrosis and emphysema (CPFE) phenotype, was identified in four patients (36.4%), of whom two had a CPFE phenotype. Desquamative interstitial pneumonia (DIP), which was smoking-related and associated with a CPFE phenotype, was observed in one patient (9.1%). Unclassifiable ILD was identified in two patients (18.2%).

The ECOG Performance Status

The ECOG performance status was as follows: An ECOG performance status of 0-1 was observed in three patients (27.3%). An ECOG score of 2 was present in five patients (45.5%). An ECOG score of 3 was observed in three patients (27.3%).

Pre-treatment Pulmonary Function

The median DLCO was 70.0% predicted (range, 22.0-84.0%). A DLCO value <40% predicted was observed in one patient (9.1%), while the remaining ten patients (90.9%) had DLCO values ≥40% predicted. The median forced expiratory volume in one second (FEV1) was 87.0% predicted (range, 51.0-99.0%), and the median forced vital capacity (FVC) was 89.0% predicted (range, 49.0-105.0%).

SABR Fractionation Regimen

SABR was delivered in five fractions in eight patients (72.7%) and in eight fractions in three patients (27.3%).

There were no missing data for any of the key clinical, functional, or dosimetric variables. Table 1 summarises the patient characteristics, ILD subtypes, and baseline lung function.

**Table 1:** Summary of key descriptive data *Worcestershire Royal Hospital **Good Hope Hospital ***Queen Elizabeth Hospital ATS/ERS: American Thoracic Society/European Respiratory Society; CTD-ILD (RA) with UIP pattern: connective tissue disease-associated interstitial lung disease-rheumatoid arthritis with usual interstitial pneumonia pattern; DIP: desquamative interstitial pneumonia; DLCO: diffusion capacity of carbon monoxide; ECOG PS: Eastern Cooperative Oncology Group performance status; FEV1: forced expiratory volume in 1 second; FVC: forced vital capacity; ILD: interstitial lung disease; ILD-GAP: interstitial lung disease Gender-Age-Physiology index; IPF (UIP pattern) with CPFE phenotype: idiopathic pulmonary fibrosis (usual interstitial pneumonia pattern) with combined pulmonary fibrosis and emphysema phenotype

Patient	Centre	Gender	ECOG PS	ATS/ERS category	Subtype (radiologic/clinicopathologic)	ILD-GAP	FVC (%)	FEV1 (%)	DLCO (%)	Dose (Gy)	Fractions
1	Worc*	Male	2	ILD with known cause/association	Exposure-related ILD (asbestos-related)	3.0	105	86	84.0	55	5
2	GHH**	Female	3	ILD with known cause/association	CTD-ILD (RA) with UIP pattern	3.0	104	92	81.0	50	5
3	QEH***	Male	1	Idiopathic interstitial pneumonia (IIP)	IPF (UIP pattern) with CPFE phenotype	3.0	89	69	70.0	50	5
4	QEH	Male	3	ILD with known cause/association	Exposure-related ILD (asbestos) with UIP pattern	3.0	98	95	63.0	60	8
5	QEH	Male	1	Unclassifiable ILD	Unclassifiable ILD	5.0	49	54	71.0	60	8
6	QEH	Male	3	Idiopathic interstitial pneumonia (IIP)	DIP (smoking-related) with CPFE phenotype	5.0	96	85	22.0	60	8
7	Solihull	Male	1	Idiopathic interstitial pneumonia (IIP)	IPF (UIP pattern) with CPFE phenotype	3.0	84	91	84.0	50	5
8	GHH	Female	2	Idiopathic interstitial pneumonia (IIP)	IPF (UIP pattern)	2.0	75	51	70.0	55	5
9	GHH	Female	2	ILD with known cause/association	CTD-ILD (RA) with UIP pattern	2.0	99	60	60.0	55	5
10	QEH	Female	2	Unclassifiable ILD	Unclassifiable ILD	4.0	93	99	45.0	50	8
11	QEH	Female	2	Idiopathic interstitial pneumonia (IIP)	IPF (UIP pattern)	4.0	63	87	67.0	50	5

Primary outcome: treatment-related toxicity

**Figure 1:**
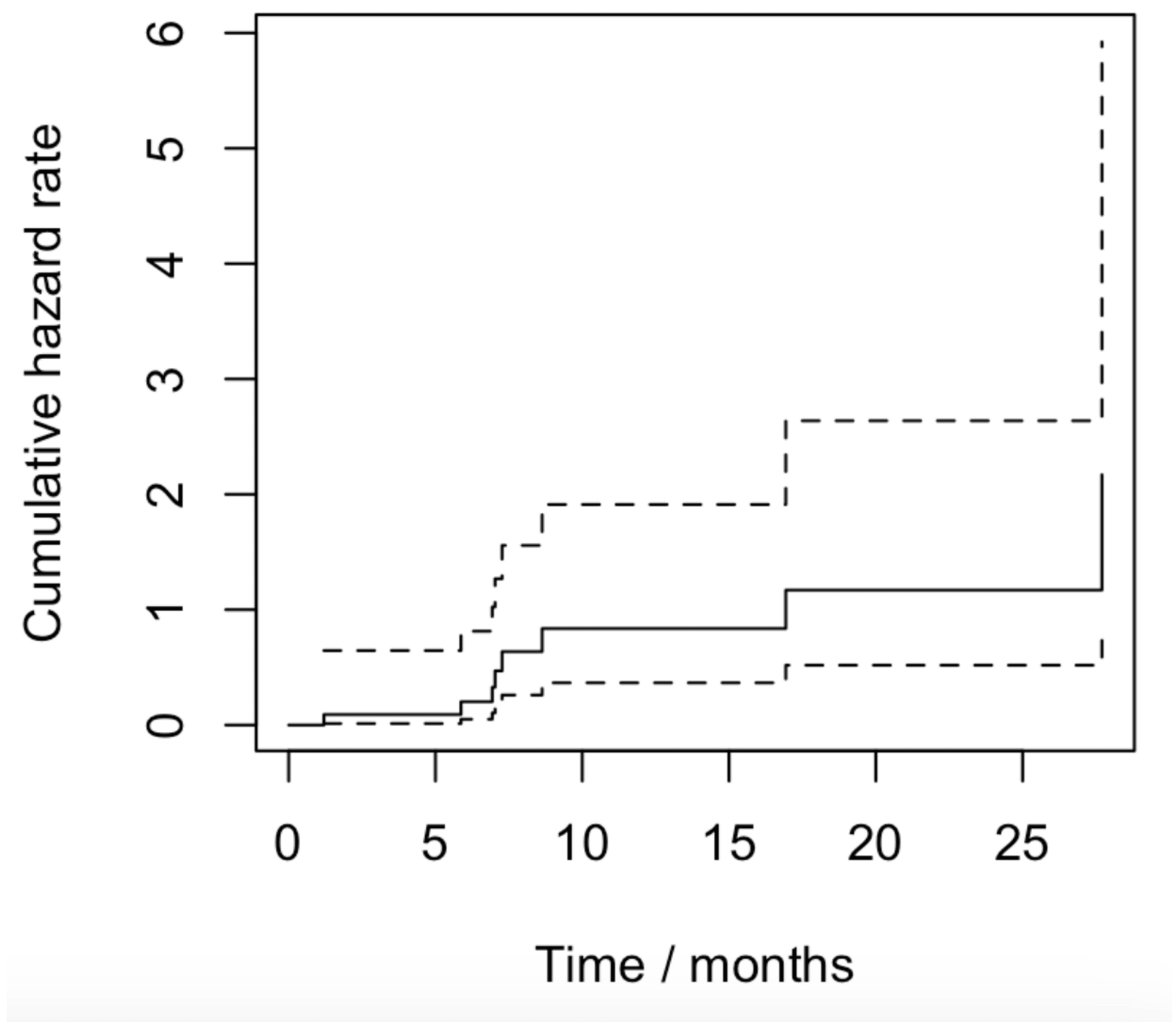
Nelson-Aalen estimator function demonstrating the toxicity hazard as a function of time

In this cohort, all patients (n = 11) experienced a SABR-related toxicity. The median time to toxicity was three months (95% CI: 1.73-3). Grade ≥3 treatment-related toxicities occurred in four of 11 patients (36.4%). Patients with a grade ≥3 toxicity had an earlier median time to toxicity of 2.2 months, with three patients dying as a result of SABR-related toxicity. Figure 1 denotes toxicity hazard as a result of SABR.

Grade 5 Toxicities (n = 3, 27.3%)

Patient 6 discontinued after two of five planned SABR fractions and died 36 days later from radiation pneumonitis with lower respiratory tract infection. Patient 7 died 97 days post-SABR from radiation pneumonitis with recurrent pneumonia. Patient 9 died 232 days post-SABR from recurrent pneumonia with concurrent pulmonary fibrosis.

Grade 3 Toxicities (n = 2, 18.2%)

Patient 5 developed decompensated heart failure 461 days post-SABR (Dmean heart 21.0 Gy; with a heart tolerance of 46 Gy). Patient 9 developed hypoxia requiring long-term oxygen therapy 162 days post-SABR.

Grades 1-2 Toxicities (n = 9, 81.8%)

Events included pulmonary fibrosis (n = 6), radiation pneumonitis (n = 3), fatigue (n = 2), pneumonia (n = 2), chest wall pain (n = 1), atelectasis (n = 1), and bronchiectasis (n = 1).

Adverse events by CTCAE v5.0 grade and time to onset are summarised in Table 2.

**Table 2:** Adverse events (CTCAE) and time to onset CTCAE: Common Terminology Criteria for Adverse Events; d: days

Patient	CTCAE 1	CTCAE 2	CTCAE 3	CTCAE 4	CTCAE 5
1	Pulmonary fibrosis - 185d	-	-	-	-
2	Atelectasis - 25d, bronchiectasis - 25d	Radiation pneumonitis - 259d	-	-	-
3	-	Pulmonary fibrosis - 508d	-	-	-
4	Pulmonary fibrosis - 90d	-	-	-	-
5	Pulmonary fibrosis -341d	-	Heart failure - 461d	-	-
6	-	-	-	-	Radiation pneumonitis - 36d, lower respiratory tract infection - 36d
7	-	-	-	-	Radiation pneumonitis - 97d, pneumonia - 97d
8	-	Pulmonary fibrosis - 94d, chest wall pain - 283d, pneumonia - 283d	-	-	-
9	-	Pulmonary fibrosis - 21d, radiation pneumonitis 107d, pneumonia - 21d + 107d	Hypoxia - 162d	-	Pneumonia - 232d
10	-	Fatigue - 75d	-	-	-
11	Radiation pneumonitis - 52d, fatigue - 52d	-	-	-	-

Secondary outcomes

Overall Survival and Progression-Free Survival (PFS)

**Figure 2:**
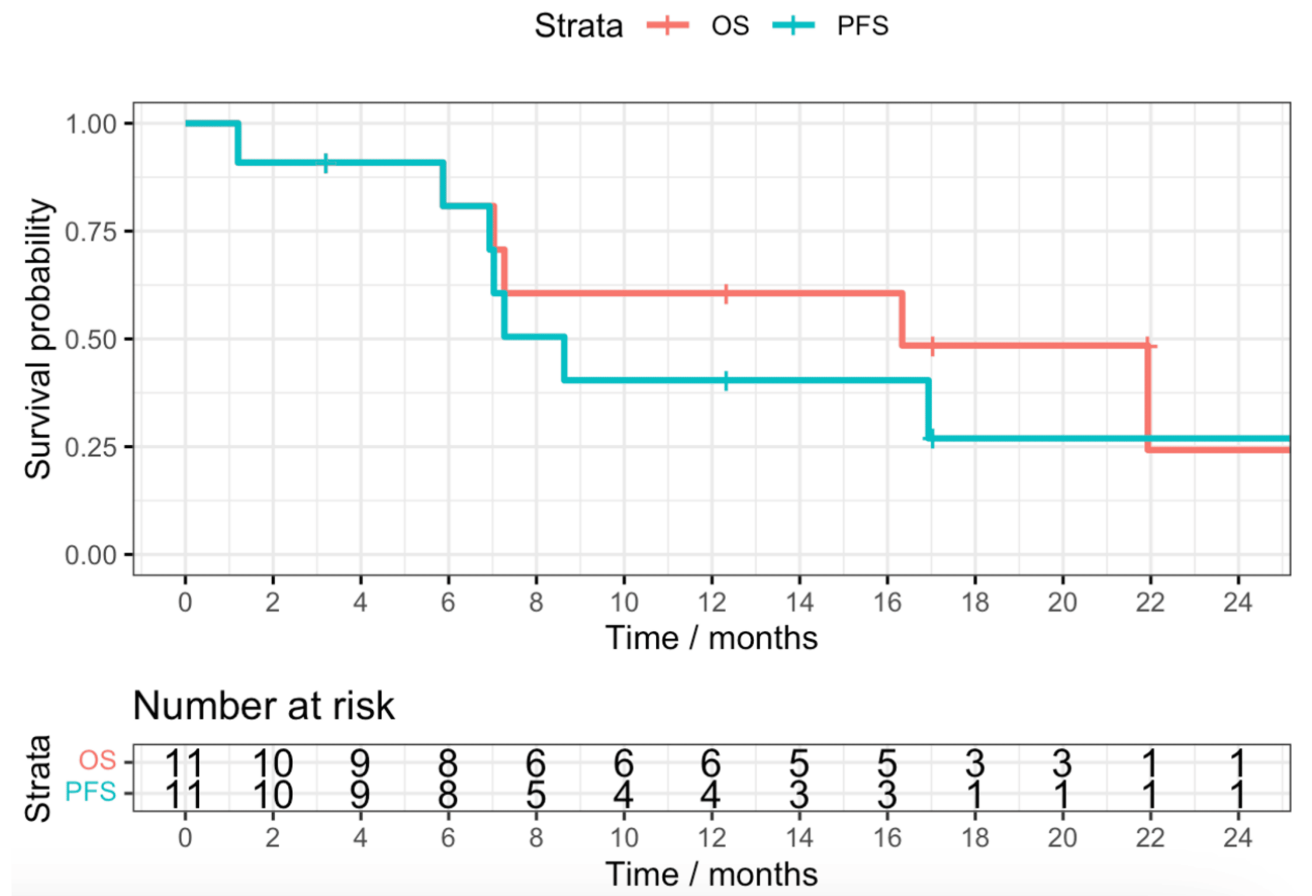
Kaplan-Meier demonstrating the progression-free survival and overall survival of the cohort

After a median follow-up time of 21.9 months (95% CI: 12.3-21.9 months), the median PFS was 8.63 months (95% CI: 6.93-8.63 months) with four of the patients having either local or metastatic progression. The median OS was 16.3 months (95% CI: 7.03-16.3 months). The predicted percentage of patients alive one year post completion of treatment was 60.6%. Figure 2 denotes the PFS and OS of the cohort.

Local Control

Local control was achieved in nine of 10 patients (90.0%), with Patient 6 excluded due to death prior to planned imaging. Local failure occurred in two patients (18.2%), both within the SABR high-dose region at 208 and 508 days, respectively.

Patterns of Progression

Four patients experienced progression of their disease. Regarding the total number of progression events, two patients experienced local recurrence, three developed regional progression involving precarinal or paratracheal lymph nodes, and one developed distant progression with brain metastases at 831 days. Progression events are summarised in Table 3, divided per patient.

**Table 3:** Progression patterns d: days

Patient no.	Local progression	Regional progression	Metastatic progression
1	-	-	Brain metastases - 831d
2	-	Precarinal lymph node progression (SCLC) - 259d	-
3	Local recurrence - 508d	Paratracheal lymph nodes - 508d	-
4	Local recurrence - 208d	Paratracheal lymph nodes - 624d	-

Subgroup and exploratory analysis

Given the small sample size (n = 11), formal multivariable modelling was not feasible. However, clinically meaningful subgroup analyses were performed to explore potential predictors of toxicity and survival.

DLCO (<40% vs. ≥40%)

One patient (9.1%) had a DLCO <40%, while 10 patients (90.9%) had a DLCO ≥40%. One grade 5 toxicity occurred in the single patient (1/1) with DLCO <40%, while two occurred in the ten patients (2/10) with DLCO ≥40%. Fisher’s exact test did not demonstrate a statistically significant association between DLCO <40% and treatment-related death.

ILD Subtype

Exposure-related ILD (asbestos-related) (n = 2; 2/11 patients): grade ≥3 toxicity was observed in two patients (0%).

CTD-ILD (RA, UIP pattern) (n = 2; 2/11 patients): grade ≥3 toxicity was observed in one of two patients (50.0%), with two events in Patient 9 (one grade 3 and one grade 5).

IPF (UIP pattern, including CPFE phenotype) (n = 4; 4/11 patients): grade ≥3 toxicity was observed in one of four patients (25.0%), consisting of one grade 5 event in Patient 7.

DIP (smoking-related, CPFE phenotype) (n = 1; 1/11 patients): grade ≥3 toxicity was observed in one patient (100%), with one grade 5 event in Patient 6.

Unclassifiable ILD (n = 2; 2/11 patients): grade ≥3 toxicity was observed in one of two patients (50.0%), with one grade 3 event in Patient 5.

When grouped by major subtype, patients with IIP (IPF including UIP pattern and CPFE phenotype, DIP smoking related to CPFE phenotype) had grade ≥3 toxicity in 40.0% (2/5) of patients. Patients with ILD with a known cause (asbestos-related, CTD-ILD (RA) with UIP pattern) had grade ≥3 toxicity in 25.0% (1/4) of patients. Patients with unclassifiable ILD had grade ≥3 toxicity in 50.0% (1/2) of patients. Fisher’s exact test did not find a statistically significant association between ILD subtype and grade ≥3 toxicity.

Radiation Parameters

The cohort had a median BED of 33.2 Gy, an upper quartile of 49.3 Gy, and a lower quartile of 30.35 Gy. The distribution of the data can be seen in Table 4. BED was stratified by: BED > median, and BED < median. Our results showed that those patients whose BED exceeded the median did not have a statistically significant increased risk of grade ≥3 toxicity, which was assessed using Fisher’s exact test.

**Table 4:** Radiation regimen per patient, α/β = 3

Patient	Dose (Gy)	Fractions	Mean Lung Dose (Gy)	EQD2 (Gy)	Mean lung dose converted to 5# equivalent plan	Mean lung dose converted to 8# per plan
1	55	5	2.1	2.9	2.1	2.4
2	50	5	4.2	6.3	4.2	4.8
3	50	5	4.4	6.5	4.4	5.1
4	60	8	4.2	5.5	3.7	4.2
5	60	8	4.4	6.5	3.8	4.4
6	60	8	2.3	2.4	2.0	2.3
7	50	5	10.2	22.5	10.2	12.0
8	55	5	5.9	9.9	5.9	6.9
9	55	5	6.7	11.8	6.7	7.8
10	50	8	4.3	5.1	3.8	4.3
11	50	5	4.8	12.5	4.8	5.6

Patients were also stratified according to the number of fractions, with 8/11 patients receiving SABR therapy delivered in five fractions (72.7%). Fisher's exact test did not demonstrate a statistically significant difference in the incidence of grade ≥3 toxicity and the fractionation schedule.

Performance Status

When stratified by ECOG performance status, patients with an ECOG of 0-1 (3/11 patients) had no statistically significant increase in OS, PFS, or grade ≥3 toxicity risk. However, patients with a worse performance status did have an estimable median OS of 16.3 months (95% CI: 7.03-16.3 months).

## Discussion

Key results

This retrospective cohort study supports the potential consideration of delivering SABR in patients with ES-NSCLC and coexisting ILD, even in a population with high-risk features such as impaired pulmonary function and predominant UIP patterns. At a median follow-up of 21.9 months (95% CI: 12.3-21.9 months), the median OS was 16.3 months (95% CI: 7.03-16.3 months) and the median PFS was 8.63 months (95% CI: 6.93-8.63 months). Local control at first planned imaging was 90.0%. The predicted percentage of patients alive one year post completion of treatment was 60.6%.

However, treatment-related toxicity was substantial: four of 11 patients (36.4%) experienced grade ≥3 toxicities, with three deaths (27.3%) attributed to radiation pneumonitis or pneumonia. Two of the three patients with a CPFE phenotype (66.7%) died within one year, although this only represented 40.0% (2/5) of IIP patients. The third death was a patient with CTD-ILD (RA) with a UIP pattern, which only represented 25.0% (1/4) of the ILD with a known cause major category. While DLCO <40% can be considered a high-risk threshold, our findings did not demonstrate a statistically significant association with treatment-related mortality in this cohort. However, low statistical power is noted due to the small sample size. These data support further prospective evaluation of modified SABR regimens in vulnerable populations.

Limitations

Several limitations constrain the generalisability and statistical robustness of this study. The small sample size (n = 11) limits statistical power, precludes multivariable modelling, and restricts inference beyond descriptive and univariable analyses. Although multicentre, as a retrospective study, it is inherently susceptible to selection bias, incomplete capture of confounders, and variability in documentation. While institutional standards were followed for imaging interpretation, SABR delivery, and toxicity grading, heterogeneity in ILD subtype diagnosis and management may persist. Furthermore, the absence of a comparator group (e.g., ILD-negative patients or alternative treatments) precludes direct comparisons of safety and efficacy. These findings should therefore be interpreted cautiously and regarded as hypothesis-generating rather than definitive.

Interpretation

This study represents, to our knowledge, the first real-world cohort analysis to evaluate SABR outcomes in patients with ES-NSCLC and coexisting ILD following the ASPIRE-ILD recommendations. While ASPIRE-ILD offered critical prospective data supporting the use of modified SABR dose constraints in a carefully selected ILD population, our cohort captures outcomes in a broader clinical setting, including patients with DLCO <40%, poorer ECOG performance status, and diverse ILD subtypes such as CPFE, that would have likely been excluded from controlled trials. Furthermore, whilst ASPIRE-ILD participants were treated with a five-fraction regimen, this cohort was treated with either five or eight fractions, taking into account more vulnerable patients with ultracentral tumour location.

Despite SABR protocols being largely aligned with ASPIRE-ILD recommendations (i.e., higher fraction regimen), treatment-related toxicity remained substantial: 36.4% of patients experienced grade ≥3 events, and 3 (27.3%) died from treatment-related causes. Although no subgroup analysis showed a significant association with severe toxicity or a change in OS, this cohort’s toxicity burden remained high. Underscoring the importance of risk stratification for physiologically impaired groups and reminding clinicians of the challenges of translating clinical trial protocols into routine practice. Furthermore, the lack of significance in a small and statistically limited cohort does not exclude the risk of morbidity or mortality. This study could have considered the utility of measuring DLCO both pre- and post-SABR as a supplementary indication of treatment-related morbidity. However, due to incomplete post-SABR data, this could not be meaningfully evaluated.

Nonetheless, median OS was 16.3 months and local control rates were 90.0%, compared to a median OS of 25 months and local control of 92% within ASPIRE-ILD [20]. Median OS in untreated NSCLC, even at early stages, was estimated at 13 months by Raz et al. [34]. This demonstrates that, with careful implementation, meaningful disease control remains achievable in real-world high-risk cohorts. Importantly, 66.7% (2/3) of fatal toxicities occurred early, within six months of treatment, and the third occurred within nine months; the first two fatalities were from radiation pneumonitis with pneumonia, and the final fatality was from pneumonia alone. Mirroring the early toxicity window reported in ASPIRE-ILD. This pattern aligns with the established temporal progression of radiation-induced lung injury after SABR, in which acute inflammatory changes such as radiation pneumonitis typically emerge within the first few months and evolve into chronic fibrotic remodelling beyond approximately six months [11,16-19,27]. Recognition of this six- to 12-month period as a critical window for post-treatment monitoring is particularly relevant for patients with coexisting ILD, who are at heightened risk of early and severe toxicity. The value of implementing a structured surveillance period following SABR should be recognised in real-world practice. 

Taken together, this data highlights the potential consideration of SABR in ILD patients with ES-NSCLC, but also the need for heightened caution in physiologically impaired patients. Future prospective studies should expand inclusion criteria to reflect real-world complexity and incorporate stratification models that integrate DLCO, ILD subtype, and performance status to optimise SABR delivery and minimise harm in this vulnerable population. Comprehensive real-world guidance should be synthesised to provide evidence-based direction on patient follow-up and risk stratification in ES-NSCLC patients with ILD undergoing SABR. Recommendations for a structured surveillance programme may include regular clinical assessments every three months during the first year post-treatment to monitor symptom burden and detect early respiratory changes. Scheduled CT imaging at approximately three to six months and again at 12 months to facilitate the identification of acute pneumonitic changes in the early phase and evolving fibrotic alterations later in the course. Pulmonary function testing, including spirometry and DLCO, to be performed at baseline, six months, and 12 months, in accordance with ATS/ERS standards [26,32,33].

Generalisability

The heterogeneous and real-world demographics within our cohort included subgroups frequently excluded from prospective trials. Notably, individuals with DLCO <40%, ECOG performance status 2-3, and diverse ILD subtypes, including CPFE and unclassifiable forms, reflecting the complexity of patients encountered in routine clinical practice. To our knowledge, this is the first report to evaluate outcomes following the implementation of ASPIRE-ILD-informed fractionation schedules within an unselected, real-world ILD population.

The design of this multicentre cohort enhances its relevance to everyday oncology practice, where rigid trial eligibility thresholds may not reflect clinical reality. Despite the modest sample size, our findings yield valuable hypothesis-generating evidence supporting the need for individualised treatment strategies in this high-risk group. Specifically, diffusion capacity, ILD subtype, and performance status should be considered in future stratification models. Validation in larger, multicentre cohorts will be essential to confirm these observations and guide risk-adapted SABR protocols in ILD-associated NSCLC.

Funding

This study received no external funding. The authors declare that no commercial or financial relationships influenced the study design, data collection, analysis, interpretation, or manuscript preparation. The research was conducted independently as part of an institutional clinical service evaluation.

## Conclusions

In contrast to prior studies, this retrospective analysis offers novel real-world insights into the delivery and outcomes of SABR for ES-NSCLC in patients with ILD, encompassing subgroups commonly excluded from clinical trials. These include individuals with severely impaired diffusion capacity (DLCO <40%), poor performance status (ECOG 2-3), and diverse ILD subtypes, including CPFE and unclassifiable ILD. To our knowledge, this is the first study to report clinical outcomes using modified SABR fractionation protocols consistent with ASPIRE-ILD recommendations in an unselected, real-world ILD population. The pragmatic cohort design improves the generalisability of findings to routine clinical practice, where patients frequently fall outside rigid trial inclusion criteria. Although limited by a small sample size, the study suggests the importance of integrating physiologic (e.g., DLCO) and clinical risk factors into SABR decision-making algorithms for this high-risk subgroup. Structured post-SABR surveillance with clinical review, interval CT imaging, and pulmonary function testing may enable early detection of pneumonitis and evolving fibrosis, improving safety in patients with ILD. Prospective, multicentre studies are warranted to validate these observations and to optimise individualised risk stratification models in ILD-associated NSCLC.

## Appendices

Appendix A 

**Table 5:** Eight-fraction organ-at-risk constraints *Reduce prescription dose target to 50 Gy if not achievable

OAR	Vol (cc)	Optimal (Gy or %)	Mandatory (Gy or %)	Achieved (Gy)
SpinalCord_PRV/Spinal_Canal	0.03	-	32.0	-
Oesophagus	0.1	-	40.0	-
BrachialPlex_L	0.1	35.0	39.0	-
BrachialPlex_R	0.1	35.0	39.0	-
Heart	0.1	40.0	46.0*	-
Trachea/ProxBronchTree	0.1	-	40.0	-
Lungs-GTV (volume = ___________cc)	V_20_	6.5%	-	-
	More than 1500 cc	Should receive <12.5Gy	-	cc^+^
	Dmean	4.5	-	-
Contralateral lung	Mean	N/A	-	-
Skin	0.1	48.0	-	-
	10.0	44.0	-	-
GreatVessels	0.1	60.0	65.0	-

Appendix B 

**Table 6:** Five-fraction organ-at-risk constraints

OAR	Vol (cc)	Optimal (Gy or %)	Mandatory (Gy or %)	Achieved (Gy)
SpinalCord_PRV/Spinal_Canal	0.03	-	25.3	-
Oesophagus	0.1	-	35.0	-
BrachialPlex_L	0.1	30.5	32.0	-
BrachialPlex_R	0.1	30.5	32.0	-
Heart	0.1	29.0	38.0	-
Trachea/ProxBronchTree	0.1	35.0	38.0	-
Lungs-GTV (volume = ___________cc)	V_20_	6.5%	-	-
	More than 1500 cc	Should receive <12.5 Gy	-	cc^+^
	Dmean	4.5	-	-
Contralateral lung	Mean	N/A	-	-
Skin	0.1	39.5	-	-
	10	36.5	-	-
Chestwall (if outlined)	0.1	43.0	-	-
